# A Review of Currently Available Fenofibrate and Fenofibric Acid Formulations

**DOI:** 10.4021/cr270w

**Published:** 2013-05-09

**Authors:** Hua Ling, John T. Luoma, Daniel Hilleman

**Affiliations:** a School of Medicine, Cardiac Center of Creighton University, Omaha, NE, USA; b Department of Cardiovascular Science, AbbVie (formerly Abbott Laboratories), North Chicago, IL, USA; c School of Pharmacy and Health Professions, Cardiac Center of Creighton University, Omaha, NE, USA

**Keywords:** Bioavailability, Fenofibrate, Fenofibric acid, Formulation, Mixed dyslipidemia, Triglycerides

## Abstract

Fenofibrate is a third-generation fibric acid derivative indicated as a monotherapy to reduce elevated low-density lipoprotein cholesterol, total cholesterol, triglycerides, and apolipoprotein B; to increase high-density lipoprotein cholesterol in patients with primary hyperlipidemia or mixed dyslipidemia; and to reduce triglycerides in patients with severe hypertriglyceridemia. In this review, the key characteristics of available fenofibrate formulations are examined. A literature search was conducted, focusing on comparative studies examining bioavailability, food effects, absorption, and lipid efficacy. Fenofibrate is highly lipophilic, virtually insoluble in water, and poorly absorbed. Coadministration with meals was necessary to maximize bioavailability of early formulations. Micronized and nanoparticle formulations of fenofibrate with reduced particle sizes were developed, resulting in greater solubility, improved bioavailability, and in some cases, the ability to be given irrespective of food. A recently introduced hydrophilic choline salt of fenofibric acid also can be taken without regard to meals, is absorbed throughout the gastrointestinal tract, has the highest bioavailability among marketed formulations, and is approved for coadministration with a statin. Differences in bioavailability of fenofibrate formulations have resulted in low-dose (40 - 67) mg and standard-dose (120 - 200 mg) formulations. Different formulations are not equivalent on a milligram-to-milligram basis. In order to prevent medication errors, resulting in underdosing or overdosing with attendant consequences, it is important for healthcare providers to recognize that the formulations of fenofibrate and fenofibric acid that are currently available vary substantially in relation to food effect, equivalency on a milligram-to-milligram basis, and indication to be coadministered with a statin.

## Introduction

Cardiovascular disease (CVD), in general, and coronary heart disease (CHD), in particular, are leading causes of death in the United States [1]. Risk factors for CHD include elevated low-density lipoprotein cholesterol (LDL-C), elevated triglycerides, and decreased concentrations of high-density lipoprotein cholesterol (HDL-C) [2]. Estimates from the National Health and Nutrition Examination Survey (NHANES) of 2003 to 2006 indicate that 53% of adults living in the United States have a lipid abnormality; 27% have high LDL-C, 23% have low HDL-C, and 30% have high triglycerides [3]. Current lipid treatment guidelines emphasize the use of LDL-C: lowering therapies for cardiovascular risk reduction. Due to the overwhelming evidence generated from outcome trials, statins, which target the rate-limiting step in cholesterol biosynthesis, remain the primary method for lowering LDL-C and reducing the incidence of cardiovascular events in these patients [2, 4].

Although statin therapy plays an important role in improving the lipid profile of patients with dyslipidemia, approximately 10% to 22% of individuals in clinical studies experience muscle pain (myalgia) during statin therapy [5]. Moreover, many patients at risk of CHD who are taking a statin are not achieving the recommended LDL-C goals. The National Cholesterol Education Program (NCEP) Evaluation ProjecT Utilizing Novel E-Technology (NEPTUNE II) Survey reported that approximately 45% of patients with diabetes mellitus and 60% with other CHD risk equivalents were not achieving the LDL-C goals established by their physicians according to NCEP Adult Treatment Panel (ATP) III, whereas 82% at very high risk of CHD were not achieving the optional NCEP ATP III LDL-C target of < 70 mg/dL [6]. Furthermore, a more recent observational study in an academic cardiology clinic found that 59.1% of patients with diabetes and established CVD did not achieve LDL-C ≤ 70 mg/dL [7]. Even when patients do reach their LDL-C goals, reduction of LDL-C alone does not always adequately reduce the risk of CVD [5, 8]. For example, 2 meta-analyses of 14 randomized clinical trials that included more than 90,000 patients showed that more than 16% of those treated with a statin experienced a CVD event over a 5-year follow-up period. The event rate was slightly higher (17%) in patients with diabetes [9, 10]. Moreover, Fruchart and colleagues reported that the percentage of residual relative risk after statin monotherapy ranges from 63% to 91% [11]. Factors contributing to residual risk include poor nutrition, lack of exercise, and failure to adequately normalize lipoproteins other than LDL-C [2]. While 2 of these residual risk factors are modifiable by lifestyle changes, an alternative therapeutic intervention strategy is sometimes necessary to correct mixed lipid abnormalities.

Mixed dyslipidemia, characterized by low levels of HDL-C and high levels of triglycerides and LDL-C [8], is highly prevalent in the general population, particularly in obese patients with metabolic syndrome and in patients with diabetes. Based on NHANES data from 2003 to 2006, it is estimated that 21% of US adults have mixed dyslipidemia, with nearly 6% having all 3 lipid abnormalities [3]. If a patient at high risk has high triglycerides or low HDL-C, consideration can be given to combining a fibrate or nicotinic acid with an LDL-C: lowering drug. When triglycerides are ≥ 200 mg/dL, non-HDL-C is a secondary target of therapy, with a goal of 30 mg/dL higher than the identified LDL-C goal [4]. Regardless of when therapy is initiated in patients with dyslipidemia, fenofibrate has been shown to significantly reduce serum total cholesterol, LDL-C, and triglycerides, and to increase HDL-C [8, 12-14].

**Figure 1:**
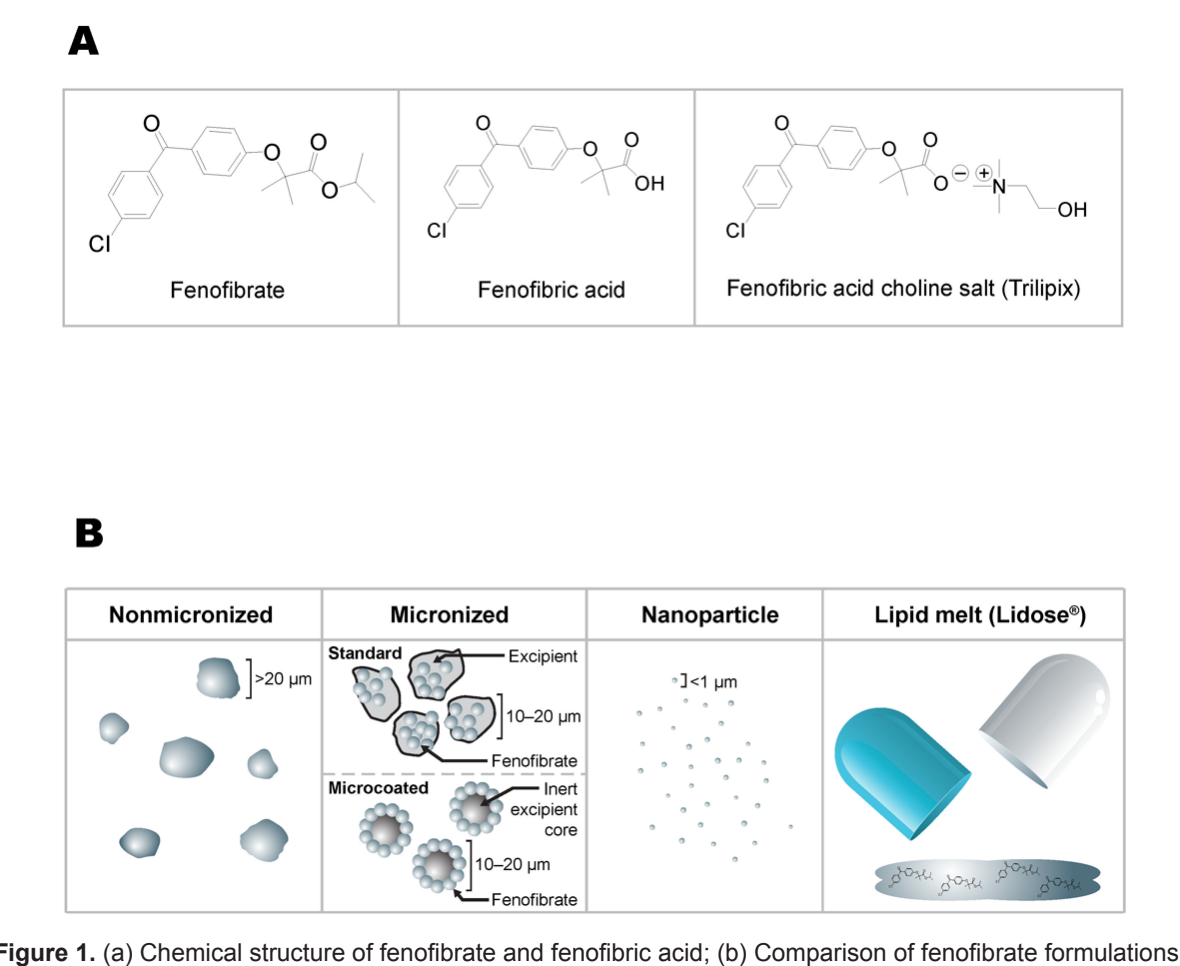
(a) Chemical structure of fenofibrate and fenofibric acid; (b) Comparison of fenofibrate formulations.

Fenofibrate acts by stimulating the activity of peroxisome proliferator-activated receptor-α (PPAR-α), a member of the PPAR subfamily of nuclear receptors that modulate the transcription of genes that regulate fatty acid and cholesterol metabolism [15]. Fenofibrate (Fig. 1a, left), a pro-drug, is pharmacologically inactive and undergoes rapid hydrolysis at the ester bond to form the active metabolite fenofibric acid (Fig. 1a, middle) [16]. However, fenofibrate is a neutral, lipophilic compound that is practically insoluble in water, making it challenging to consistently achieve therapeutic levels [17]. Thus, several different formulations of fenofibrate have been developed in an attempt to increase its overall solubility since its introduction in the United States in 1998. In this review, we compare the key, clinically relevant characteristics of the fenofibrate formulations that are currently available, specifically focusing on comparative studies examining the bioavailability, food effects, and absorption of these fenofibrate formulations.

## Methods

An electronic search of the scientific literature was carried out with PubMed (pubmed) using the following string: ((formulation OR formulations OR bioequivalence OR bioavailability) AND (fenofibrate OR fenofibric acid)) OR ((safety OR efficacy) AND (fenofibrate OR fenofibric acid) AND statins) OR ((nanotechnology OR nanoparticles OR micronized OR microparticles) AND (improved therapeutic outcomes)). Results were limited to English language, Clinical Trials, Humans, Reviews, and publication date between 1993 and 2012, yielding 46 citations of interest. Of these 46 citations, only clinical trials that directly compared one fenofibrate formulation against another, explored the food effects of fenofibrate, or examined coadministration of a fibrate with a statin were included, yielding a total of 27 articles, which formed the basis of this review. Additional sources included package inserts for all drugs discussed and meeting abstracts when primary manuscripts with pertinent information were unavailable.

## Results

### Fenofibrate formulations currently available

Challenges with solubility led to the development of novel fenofibrate formulations (Table 1) intended to increase the overall bioavailability via several different approaches (Fig 1b) [17, 18]. Initially, micronized formulations increased solubility by reducing particle size and increasing surface area [19]. Later, tablets combining the classic micronization process with a microcoating network of hydrophilic polyvinylpyrrolidone led to increased dissolution rates and greater bioavailability [20]. Subsequently, insoluble drug delivery^®^ microparticle (IDD-P) tablets that used phospholipid agents that modify surface properties to prevent reaggregation were developed, thus preserving the expanded drug surface area of microparticles while accelerating dissolution, resulting in better absorption [21]. Nanoparticle formulations further reduced particle size, leading to a significantly increased ratio of surface area to volume and greater bioavailability (Fig. 1b) [18]. Finally, a formulation of the active metabolite (fenofibric acid) in a choline salt form was developed, generating a hydrophilic compound with the greatest bioavailability of the available formulations (Fig. 1a, right) [22].

**Table 1:** Fenofibrate and Fenofibric Acid Formulations Currently Available

Trade Name (Company)	Form and Doses, mg	Food Effect	Equivalence	Statin Coadministration
Non-micronized fenofibrate				
Fenoglide [25] (Santarus)	Tablet 40, 120	Yes	3 × 40 mg or single 120 mg equivalent to 130 mg fenofibrate capsule (high-fat fed conditions)	Not approved
Lipofen [24] (Kowa)	Capsule (Lidose) 50, 150	Yes	150 mg equivalent to 160 mg Tricor (low-fat and high-fat fed conditions)	Not approved
Lofibra [23] (Gate)	Tablet 54, 160	Yes	54 mg equivalent to 67 mg fenofibrate tablet; 160 mg equivalent to 200 mg fenofibrate capsule (fed conditions)	Not approved
Triglide [27] (Shionogi)	Tablet (IDD-P) 50, 160	Yes	160 mg equivalent to 200 mg micronized fenofibrate capsule (low-fat fed conditions), but absorption rate is 32% faster	Not approved
Fenofibrate [30] (Global Pharmaceutical)	Tablet 54, 160	Yes	3 × 48 mg or single 145 mg equivalent to 200 mg fenofibrate capsule (fed conditions)	Not approved
Fenofibrate [31] (Mylan)	Tablet 54, 160	Yes	54 mg equivalent to 67 mg fenofibrate capsule; 160 mg equivalent to 200 mg fenofibrate capsule (fed conditions)	Not approved
Fenofibrate [32] (Ranbaxy Pharmaceuticals)	Tablet 54, 160	Yes	54 mg equivalent to 67 mg fenofibrate capsule; 160 mg equivalent to 200 mg fenofibrate capsule (fed conditions)	Not approved
Micronized fenofibrate				
Antara [29] (Lupin)	Capsule 43, 130	No	130 mg equivalent to 200 mg fenofibrate capsule (low-fat fed conditions)	Not approved
Tricor [33] (Abbott Laboratories)	Capsule 67, 200	Yes	No data	Not approved
Lofibra [23] (Gate)	Capsule 67, 134, 200	Yes	67 mg equivalent to 100 mg non-micronized fenofibrate	Not approved
Fenofibrate-micronized [34] (Global Pharmaceutical)	Capsule 67, 134, 200	Yes	67 mg equivalent to 100 mg non-micronized fenofibrate	Not approved
Nanocrystal fenofibrate				
Tricor [35] (Abbott)	Tablet 48, 145	No	3 × 48 mg or 145 mg equivalent to 200 mg micronized fenofibrate capsule (fed conditions)	Not approved
Fenofibric acid				
Trilipix [22] (Abbott)	Capsule (delayed release) 45, 135	No	135 mg equivalent to 200 mg micronized fenofibrate capsule (fed conditions)	Approved

IDD-P: insoluble drug delivery microparticle.

Despite these advancements, adequate absorption of many marketed fenofibrate formulations still requires the presence of food, particularly a high-fat meal [21]. In general, fenofibrate products can be grouped with regard to their food effects. For example, fenofibrate formulations that should be taken with meals include non-micronized tablets (Fenoglide^®^, Lofibra^®^, generic), micronized capsules (Lofibra^®^ and generic), microcoated micronized tablets (Lofibra^®^), and fenofibrate hard gelatin capsules (Lipofen^®^) [23-26]. Fenofibrate formulations that can be taken with or without meals include nanoparticle tablets (Tricor^®^), IDD-P tablets (Triglide^®^), micronized capsules (Antara^®^), and the choline salt of fenofibric acid (Trilipix^®^) (Table 1) [22, 27-29].

### Comparative clinical trials of fenofibrate formulations

#### Bioavailability and food effects

Since fenofibrate was first introduced, the main limitation has been the low bioavailability when taken orally, especially without a high-fat meal. In the presence of food, fenofibrate absorption from microcoated tablets increases up to 35% [36]. Partly because it is desirable to limit high-fat meals in patients with hypertriglyceridemia, for which fenofibrate is often prescribed [2](2), studies of newer formulations of fenofibrate have examined whether these drugs can be taken without food. A number of these studies made pharmacokinetic comparisons among fenofibrate products under fasting, low-fat fed, and high-fat fed conditions.

Guivarc’h and colleagues compared the bioavailability and food effects of 3 fenofibrate formulations: IDD-P fenofibrate (160 mg tablets), microcoated fenofibrate (MCF; 160 mg tablets; ie, Tricor^®^), and micronized fenofibrate (MF; 200 mg capsules; ie, Tricor^®^ and Lofibra^®^) [21]. Based on 6 single-dose pharmacokinetic studies in healthy volunteers, IDD-P fenofibrate was bioequivalent under fasting, low-fat fed, and high-fat fed conditions (90% confidence interval (CI)) for ratios of area under the plasma concentration–time curve within the 80-125% US Food and Drug Administration (FDA) guidelines for bioequivalence), whereas the bioavailability of MCF and MF were noticeably greater under high-fat fed conditions [21]. Under low-fat fed conditions, all 3 formulations were bioequivalent (90% CIs within the 80-125% bioequivalence range). In contrast, during fasting conditions, IDD-P fenofibrate had a significantly higher bioavailability than MF (42.56% (90% CI, 27.29-59.65%]; P < 0.001). Microcoated fenofibrate tablets had generally comparable bioavailability to that of MF capsules under fasting conditions, although the upper portion of the 90% CI exceeded 125% (133%). Although all 3 fibrate formulations were similarly well tolerated, these studies clearly show that they are not bioequivalent when dosed with a high-fat meal and, in some cases, on an empty stomach.

The Lidose^®^ drug delivery technology uses a lipid excipient mixture to increase fenofibrate bioavailability by about 25% compared with the micronized form [37]. However, the greater bioavailability obtained with the Lidose^®^ technology relative to MF may be compromised by food effects. Absorption of a fenofibrate formulation (Lipofen^®^; Fig. 1b) that employs this technology was increased by 25% when taken with a low-fat meal and by 58% with a high-fat meal when compared with fasting conditions [24]. When the bioequivalence of Lipirex^®^, another fenofibrate formulation that uses the Lidose^®^ technology (available outside the United States), and an MF formulation (67 and 200 mg) were compared in an open-label, crossover study in healthy volunteers, these formulations of fenofibrate were bioequivalent on a milligram-to-milligram basis under fed conditions [38]. Thus, formulations with Lidose^®^ technology may have greater bioavailability than MF under certain conditions, but not others.

Limited evidence suggests that generic fenofibrate products with the same formulation may be bioequivalent under fasting conditions. The relative bioavailability of Lipivim^®^ (a Romanian generic fenofibrate) with respect to an unnamed marketed fenofibrate was determined in a single-dose (200 mg), randomized, crossover study in 24 healthy volunteers dosed without food. The 90% CIs of the mean ratios of the plasma pharmacokinetic variables were within the conventional bioequivalence range of 80% to 125% [39]. Both fibrates were equally well tolerated. This study suggests that when formulations are bioequivalent, a generic may be substituted for a name brand formulation, as long as the doses are taken without food.

Alterations in bioequivalence can occur not only because of diet, but also due to inherent variations in absorption within the gastrointestinal tract. Zhu and colleagues compared the gastrointestinal absorption characteristics and absolute bioavailability of fenofibric acid (130 mg oral suspension) and nanocrystal fenofibrate (145 mg oral suspension) administered at least 4 hours after a light breakfast in healthy volunteers [40]. Although both fibrates were well tolerated, the bioavailability of fenofibric acid and fenofibrate differed in the stomach (81% vs 69%, respectively; P *=* 0.063), proximal small bowel (88% vs 73%; P *=* 0.075), distal small bowel (84% vs 66%; P*=* 0.033), and colon (78% vs 22%; P *<* 0.0001). These results show that orally administered fenofibric acid has a significantly higher bioavailability than fenofibrate in 2 out of 4 sections of the gastrointestinal tract, suggesting that the fenofibric acid formulation has improved absorption characteristics compared with the nanocrystal fenofibrate formulation. Thus, plasma exposure with the fenofibric acid formulation would be expected to be greater than with the nanocrystal fenofibrate formulation regardless of individual differences in the degree of motility in separate segments of the gastrointestinal tract.

No studies have directly compared the bioequivalence of the currently available doses of fenofibric acid to other fibrate formulations; however, the pharmacokinetics of fenofibric acid (Trilipix^®^; 135 mg) [22] can be compared indirectly with the nanoparticle formulation of fenofibrate tablets (Tricor^®^; 145 mg) [28, 35] using results from 2 separate studies with identical designs and a common reference compound (200 mg MF capsule). Although both formulations met bioequivalence criteria with their respective reference compound, after normalization, the fenofibric acid 135 mg formulation produced a 4.4% higher fenofibric acid exposure and an 11.3% lower maximum concentration than the fenofibrate 145 mg compound [40]. These studies are consistent with the findings of Zhu and colleagues from the gastrointestinal study discussed previously, which demonstrated that fenofibric acid had greater absorption versus fenofibrate throughout the gastrointestinal tract and has improved bioavailability.

An article by Godfrey and colleagues described the bioequivalence of a fenofibrate 145 mg formulation with a lower-dose fenofibric acid formulation available outside the United States (fenofibric acid 105 mg) [41]. In 2 studies, healthy volunteers were administered a single oral dose of fenofibric acid or fenofibrate under fasting or fed conditions. The 90% CIs were within the 80% to 125% limits for pharmacokinetic parameters both in fasted and fed conditions, demonstrating bioequivalence for these doses.

These studies underscore the clinical relevance of the effects of food and bioavailability for treatment strategies that incorporate fibrate and fenofibric acid products. It is important to note that differences in food effects seen with various fenofibrate and fenofibric acid formulations can alter a patient’s adherence to treatment. For example, patients who take other medications that require dosing with food may confuse the fenofibrate dosing with their other medications, potentially reducing bioavailability. Moreover, adhering to a low-fat diet as part of lipid-modifying therapy [2, 42]may compromise fenofibrate bioavailability in some currently available fenofibrate formulations. Assuming patients and prescribers are aware of the effect of food on drug absorption, the requirement to take doses with meals leads to added inconvenience and complexity, which could diminish adherence to fenofibrate treatment. Together, the complications introduced by food and fat effects on bioavailability of currently marketed fenofibrate products emphasize the importance of careful consideration of consequences when switching patients from one fenofibrate formulation to another.

#### Lipid-lowering properties

Although non-adherence to the food and fat requirements seen with currently marketed fenofibrate formulations can clearly lead to inconsistent, unpredictable, and suboptimal pharmacokinetics, few studies have actually compared the influence of food effects on the lipid-lowering efficacy of these products. Davidson and colleagues performed a retrospective analysis of lipid levels for patients with a diagnosis of hypertension, dyslipidemia, or diabetes mellitus who switched from standard MCF 160 mg (requiring dosing with food) to the 145 mg nanoparticle tablet (no food effect) to determine the effects of food on the overall lipid response [43]. Statistically significant decreases (P ≤ 0.004) were observed for total cholesterol, LDL-C, and triglycerides in patients who switched from fenofibrate 160 mg (food effects) to fenofibrate 145 mg (no food effects). The authors concluded that patients treated in an outpatient clinical practice would have better lipid results if prescribed a fenofibrate that has a less restrictive dosing regimen.

Similarly, a study conducted by Maciejewski and Hilleman compared the effectiveness of the same microcoated 160 mg tablet formulation with the same 145 mg nanoparticle tablet formulation in patients with CHD and dyslipidemia [44]. Patients (N = 130) were treated for a minimum of 6 months with fenofibrate 160 mg and were switched to treatment with fenofibrate 145 mg for a minimum of 3 months. Statistically significant reductions of 4.6% to 5.1% for mean triglycerides and 2.3% to 2.8% for mean LDL-C were observed after switching from fenofibrate 160 mg to fenofibrate 145 mg (P ≤ 0.002). Although no significant changes in the level of HDL-C were observed, the improvements in LDL-C and triglycerides indicate that the nanoparticle fenofibrate formulation was more effective for treating mixed dyslipidemia. Although elimination of a positive food effect simplifies dosing, it is still unclear whether the improvements in lipid parameters were due to increased bioavailability or increased patient compliance.

Although the studies just described clearly show the consequence of food on plasma lipid parameters, interestingly, the package insert for one formulation of MF (Antara^®^ capsules) states that it may be taken without regard to meals [29]. There is evidence that this product is better absorbed with a high-fat meal; when administered with a high-fat meal, there was a 26% increase in fenofibric acid bioavailability and a 108% increase in maximum concentration compared with the fasting state [29]. Despite the pharmacokinetic implications of taking Antara^®^ with a high-fat meal, data from a clinical study showed comparable outcomes on serum triglycerides and cholesterol concentrations when Antara^®^ (130 mg) was taken once daily with or between meals [45]. Thus, although this study suggested differences in bioequivalence under the fasting and fed states, efficacy was less affected by administration with food and formed the basis for the indication to administer the drug without regard to meals.

#### Coadministration with a statin

The addition of a fibrate to statin therapy offers the potential to substantially lower LDL-C and triglycerides and to elevate HDL-C. To date, only fenofibric acid (Trilipix^®^) has been approved by the FDA for coadministration with a statin [22]. In the past, warnings concerning an increased risk of toxicity and cases of myopathy and rhabdomyolysis associated with the combination of a fibrate and a statin have limited their combined use [13]; however, long-term studies with fenofibrate or fenofibric acid and statins have demonstrated that this combination is generally well tolerated [33, 46-50]. Several 12-week controlled studies of a combination of fenofibric acid with atorvastatin, rosuvastatin, or simvastatin therapy have demonstrated significant improvements in LDL-C, HDL-C, triglycerides, and non-HDL-C levels compared with prespecified monotherapies [51-55]. These benefits were maintained for up to 2 years in an open-label extension study of the combination therapies [56]. Improvements also were evident with combination therapy in a post hoc analysis of patients with type 2 diabetes mellitus [57] who comprised subsets of the populations in two 12-week controlled studies mentioned previously [52, 55]. Another post hoc analysis of data from a 52-week extension study demonstrated lipid improvements in a subpopulation of patients with LDL-C < 100 mg/dL, yet persistently elevated triglycerides (> 200 mg/dL), after 12 weeks of statin monotherapy during three 12-week controlled trials [58]. In all of the studies, safety profiles were similar between combination treatments and monotherapies. Despite the promise of these combination therapies, studies examining the use of fenofibrate or fenofibric acid with high-dose statin therapy have yet to be completed, and at least for now, these combinations should be avoided.

Because of the clear efficacy of combination therapy for treating mixed dyslipidemia, Veloxis Pharmaceuticals is developing a fixed-dose combination tablet of fenofibrate (100 mg) and atorvastatin (40 mg; LCP-AtorFen^®^). Short-term efficacy and safety studies suggest that AtorFen^®^ generally led to greater decreases in triglycerides and greater increases in HDL-C than either atorvastatin or fenofibrate alone [59]. A 12-month open-label extension study was initiated in 2007 (NCT00664859); however, the current status of this trial is unknown. Thus, long-term efficacy and safety studies are still necessary to establish the benefits of this particular combination drug.

## Discussion

Although statins are effective at lowering LDL-C, their efficacy on other lipid targets, specifically HDL-C and triglycerides, is less substantial. Continued abnormalities in these lipids despite optimal statin monotherapy may contribute to residual CVD risk. Combining fenofibrate with a statin is a therapeutic option to address these additional lipid abnormalities. Although evidence clearly illustrates that fenofibrate, alone or in combination with statins, reduces plasma levels of LDL-C and triglycerides, raises HDL-C levels, and improves other CVD risk factors [8, 12-14], the ACCORD and FIELD trials raised concerns that fenofibrate may provide little additional benefit, in terms of cardiovascular event reduction, in patients without atherogenic dyslipidemia [33, 46]. However, subsequent meta-analyses (which incorporated these same studies) have highlighted the lipid-modifying benefit of fibrate treatment in patients with elevated triglycerides and low HDL-C [60-62].

The most recent of these meta-analyses, which included 5 trials of almost 4,700 patients with low HDL-C and high triglycerides, concluded that fibrate therapy resulted in a significant reduction in cardiovascular events in patients with hypertriglyceridemia alone, low HDL-C levels alone, or both (28%, 17%, and 30%, respectively) [62]. This effect was minimal in patients with normal HDL-C, triglycerides, or both (reductions of 3%, 6%, and 6%, respectively). Although these data point to the potential benefit of fenofibrate treatment in the subpopulation with mixed dyslipidemia, no published clinical study to date has tested the effect. In the future, it will be important to compare the bioavailability and overall efficacy of different fenofibrate formulations in this subset of individuals.

Together, the comparative clinical trials in this review highlight the importance of the relationship between fenofibrate formulation and bioequivalence, food effects, and overall efficacy. Bioequivalence has not been consistent for different fenofibrate formulations and doses, particularly when food effects are observed. However, even when a food effect is not evident, absorption can be affected by differences in gastrointestinal transit and residence times. Although some fenofibrate treatment regimens still require dosing with food, finding a formulation that can be administered independently may provide greater convenience for patients. The elimination of a positive food effect and the resultant simplification of the dosing regimen may increase patient compliance, and thus, overall treatment effectiveness.

In order to prevent medication errors that may result in underdosing or overdosing, with attendant consequences, it is important for healthcare providers to recognize that various formulations of fenofibrate and fenofibric acid that are currently available vary substantially in relation to food effect, equivalency on a milligram-to-milligram basis, and indication to be coadministered with a statin. Except for generic equivalents of a specific branded product, fenofibrate formulations are not bioequivalent, and should not be treated as such.

## References

[1] Rosamond W, Flegal K, Furie K, Go A, Greenlund K, Haase N, Hailpern SM (2008). Heart disease and stroke statistics—2008 update: a report from the American Heart Association Statistics Committee and Stroke Statistics Subcommittee. Circulation.

[2] (2002). Third Report of the National Cholesterol Education Program (NCEP) Expert Panel on Detection, Evaluation, and Treatment of High Blood Cholesterol in Adults (Adult Treatment Panel III) final report.

[3] Toth PP, Potter D, Ming EE (2012). Prevalence of lipid abnormalities in the United States: the National Health and Nutrition Examination Survey 2003-2006. J Clin Lipidol.

[4] Grundy SM, Cleeman JI, Merz CN, Brewer HB, Clark LT, Hunninghake DB, Pasternak RC (2004). Implications of recent clinical trials for the National Cholesterol Education Program Adult Treatment Panel III guidelines. Circulation.

[5] Fernandez G, Spatz ES, Jablecki C, Phillips PS (2011). Statin myopathy: a common dilemma not reflected in clinical trials. Cleve Clin J Med.

[6] Davidson MH, Maki KC, Pearson TA, Pasternak RC, Deedwania PC, McKenney JM, Fonarow GC (2005). Results of the National Cholesterol Education (NCEP) Program Evaluation ProjecT Utilizing Novel E-Technology (NEPTUNE) II survey and implications for treatment under the recent NCEP Writing Group recommendations. Am J Cardiol.

[7] DeGuzman PB, Akosah KO, Simpson AG, Barbieri KE, Megginson GC, Goldberg RI, Beller GA (2012). Sub-optimal achievement of guideline-derived lipid goals in management of diabetes patients with atherosclerotic cardiovascular disease, despite high use of evidence-based therapies. Diab Vasc Dis Res.

[8] Saurav A, Kaushik M, Mohiuddin SM (2012). Fenofibric acid for hyperlipidemia. Expert Opin Pharmacother.

[9] Baigent C, Keech A, Kearney PM, Blackwell L, Buck G, Pollicino C, Kirby A (2005). Efficacy and safety of cholesterol-lowering treatment: prospective meta-analysis of data from 90,056 participants in 14 randomised trials of statins. Lancet.

[10] Kearney PM, Blackwell L, Collins R, Keech A, Simes J, Peto R, Armitage J (2008). Efficacy of cholesterol-lowering therapy in 18,686 people with diabetes in 14 randomised trials of statins: a meta-analysis. Lancet.

[11] Fruchart JC, Sacks F, Hermans MP, Assmann G, Brown WV, Ceska R, Chapman MJ (2008). The Residual Risk Reduction Initiative: a call to action to reduce residual vascular risk in patients with dyslipidemia. Am J Cardiol.

[12] Filippatos TD, Elisaf MS (2011). Fenofibrate plus simvastatin (fixed-dose combination) for the treatment of dyslipidaemia. Expert Opin Pharmacother.

[13] Schima SM, Maciejewski SR, Hilleman DE, Williams MA, Mohiuddin SM (2010). Fibrate therapy in the management of dyslipidemias, alone and in combination with statins: role of delayed-release fenofibric acid. Expert Opin Pharmacother.

[14] Keating GM, Croom KF (2007). Fenofibrate: a review of its use in primary dyslipidaemia, the metabolic syndrome and type 2 diabetes mellitus. Drugs.

[15] Staels B, Dallongeville J, Auwerx J, Schoonjans K, Leitersdorf E, Fruchart JC (1998). Mechanism of action of fibrates on lipid and lipoprotein metabolism. Circulation.

[16] Adkins JC, Faulds D (1997). Micronised fenofibrate: a review of its pharmacodynamic properties and clinical efficacy in the management of dyslipidaemia. Drugs.

[17] Vogt M, Kunath K, Dressman JB (2008). Dissolution enhancement of fenofibrate by micronization, cogrinding and spray-drying: comparison with commercial preparations. Eur J Pharm Biopharm.

[18] Bosselmann S, Williams RO (2012). Has nanotechnology led to improved therapeutic outcomes?. Drug Dev Ind Pharm.

[19] Rawat N, Kumar SM, Mahadevan N (2011). Solubility: particle size reduction is a promising approach to improve the bioavailability of lipophillic drugs. Int J Recent Adv Pharm Res.

[20] Guichard JP, Blouquin P, Qing Y (2000). A new formulation of fenofibrate: suprabioavailable tablets. Curr Med Res Opin.

[21] Guivarc'h PH, Vachon MG, Fordyce D (2004). A new fenofibrate formulation: results of six single-dose, clinical studies of bioavailability under fed and fasting conditions. Clin Ther.

[22] Trilipix® (fenofibric acid delayed release capsules). Full Prescribing Information, Abbott Laboratories, North Chicago, IL, 2011

[23] Lofibra® (fenofibrate tablets). Full Prescribing Information, Gate Pharmaceuticals, Sellersville, PA, 2010

[24] Lipofen® (fenofibrate). Full Prescribing Information, Galephar Pharmaceutical Research, Juncos, PR, 2007

[25] Fenoglide (fenofibrate). Full Prescribing Information, Sciele Pharma, Atlanta, GA, 2009

[26] Lofibra® (fenofibrate capsules). Full Prescribing Information, Gate Pharmaceuticals, Sellersville, PA, 2003

[27] Triglide® (fenofibrate). Full Prescribing Information, Sciele Pharma, Atlanta, GA, 2007

[28] Tricor® (fenofibrate). Full Prescribing Information, Abbott Laboratories, Chicago, IL, 2004

[29] Antara® (fenofibrate). Full Prescribing Information, Oscient Pharmaceuticals Corporation, Emeryville, CA, 2006

[30] Fenofibrate (generic). Full Prescribing Information, Global Pharmaceuticals, Philadelphia, PA, 2011

[31] Fenofibrate (generic). Full Prescribing Information, Mylan Pharmaceuticals Inc, Morgantown, WV, 2010

[32] Fenofibrate (generic). Full Prescribing Information, Ranbaxy Pharmaceuticals Inc, Jacksonville, FL, 2009

[33] Keech A, Simes RJ, Barter P, Best J, Scott R, Taskinen MR, Forder P (2005). Effects of long-term fenofibrate therapy on cardiovascular events in 9795 people with type 2 diabetes mellitus (the FIELD study): randomised controlled trial. Lancet.

[34] Micronized-Fenofibrate (generic). Full Prescribing Information, Global Pharmaceuticals, Philadelphia, PA, 2009

[35] Tricor® (fenofibrate). Full Prescribing Information, Abbott Laboratories, Chicago, IL, 2011

[36] Sauron R, Wilkins M, Jessent V, Dubois A, Maillot C, Weil A (2006). Absence of a food effect with a 145 mg nanoparticle fenofibrate tablet formulation. Int J Clin Pharmacol Ther.

[37] Armenti JBV (2010). Handbook of Drug-Nutrient Interactions.

[38] Sonet B, Vanderbist F, Streel B, Houin G (2002). Randomised crossover studies of the bioequivalence of two fenofibrate formulations after administration of a single oral dose in healthy volunteers. Arzneimittelforschung.

[39] Vlase L, Popa A, Muntean D, Leucuta SE (2010). Pharmacokinetics and comparative bioavailability of two fenofibrate capsule formulations in healthy volunteers. Arzneimittelforschung.

[40] Zhu T, Ansquer JC, Kelly MT, Sleep DJ, Pradhan RS (2010). Comparison of the gastrointestinal absorption and bioavailability of fenofibrate and fenofibric acid in humans. J Clin Pharmacol.

[41] Godfrey AR, Digiacinto J, Davis MW (2011). Single-dose bioequivalence of 105-mg fenofibric acid tablets versus 145-mg fenofibrate tablets under fasting and fed conditions: a report of two phase I, open-label, single-dose, randomized, crossover clinical trials. Clin Ther.

[42] (2001). Executive Summary of The Third Report of The National Cholesterol Education Program (NCEP) Expert Panel on Detection, Evaluation, And Treatment of High Blood Cholesterol In Adults (Adult Treatment Panel III).

[43] Davidson MH, Jones PH (2008). Retrospective comparison of the effectiveness of a fenofibrate 145 mg formulation compared with the standard 160 mg tablet. Clin Drug Investig.

[44] Maciejewski S, Hilleman D (2008). Effectiveness of a fenofibrate 145-mg nanoparticle tablet formulation compared with the standard 160-mg tablet in patients with coronary heart disease and dyslipidemia. Pharmacotherapy.

[45] Davidson MH, Bays H, Rhyne J, Stein E, Rotenberg K, Doyle R (2005). Efficacy and safety profile of fenofibrate-coated microgranules 130 mg, with and without food, in patients with hypertriglyceridemia and the metabolic syndrome: an 8-week, randomized, double-blind, placebo-controlled study. Clin Ther.

[46] Ginsberg HN, Elam MB, Lovato LC, Crouse JR, Leiter LA, Linz P, Friedewald WT (2010). Effects of combination lipid therapy in type 2 diabetes mellitus. N Engl J Med.

[47] Athyros VG, Papageorgiou AA, Athyrou VV, Demitriadis DS, Kontopoulos AG (2002). Atorvastatin and micronized fenofibrate alone and in combination in type 2 diabetes with combined hyperlipidemia. Diabetes Care.

[48] Durrington PN, Tuomilehto J, Hamann A, Kallend D, Smith K (2004). Rosuvastatin and fenofibrate alone and in combination in type 2 diabetes patients with combined hyperlipidaemia. Diabetes Res Clin Pract.

[49] Koh KK, Quon MJ, Han SH, Chung WJ, Ahn JY, Seo YH, Choi IS (2005). Additive beneficial effects of fenofibrate combined with atorvastatin in the treatment of combined hyperlipidemia. J Am Coll Cardiol.

[50] Bays HE, Jones PH, Mohiuddin SM, Kelly MT, Sun H, Setze CM, Buttler SM (2008). Long-term safety and efficacy of fenofibric acid in combination with statin therapy for the treatment of patients with mixed dyslipidemia. J Clin Lipidol.

[51] Goldberg AC, Bays HE, Ballantyne CM, Kelly MT, Buttler SM, Setze CM, Sleep DJ (2009). Efficacy and safety of ABT-335 (fenofibric acid) in combination with atorvastatin in patients with mixed dyslipidemia. Am J Cardiol.

[52] Jones PH, Davidson MH, Kashyap ML, Kelly MT, Buttler SM, Setze CM, Sleep DJ (2009). Efficacy and safety of ABT-335 (fenofibric acid) in combination with rosuvastatin in patients with mixed dyslipidemia: a phase 3 study. Atherosclerosis.

[53] Mohiuddin SM, Pepine CJ, Kelly MT, Buttler SM, Setze CM, Sleep DJ, Stolzenbach JC (2009). Efficacy and safety of ABT-335 (fenofibric acid) in combination with simvastatin in patients with mixed dyslipidemia: a phase 3, randomized, controlled study. Am Heart J.

[54] Roth EM, Rosenson RS, Carlson DM, Fukumoto SM, Setze CM, Blasetto JW, Khurmi NS (2009). A phase III study evaluating the efficacy and safety of 135 mg fenofibric acid (ABT-335) in combination with 5 mg rosuvastatin in patients with atherogenic dyslipidemia. J Am Coll Cardiol.

[55] Roth EM, Rosenson RS, Carlson DM, Fukumoto SM, Setze CM, Blasetto JW, Khurmi NS (2010). Efficacy and safety of rosuvastatin 5 mg in combination with fenofibric acid 135 mg in patients with mixed dyslipidemia - a phase 3 study. Cardiovasc Drugs Ther.

[56] Kipnes MS, Roth EM, Rhyne JM, Setze CM, Lele A, Kelly MT, Sleep DJ (2010). Year two assessment of fenofibric acid and moderate-dose statin combination: a phase 3, open-label, extension study. Clin Drug Investig.

[57] Rosenson RS, Carlson DM, Kelly MT, Setze CM, Hirshberg B, Stolzenbach JC, Williams LA (2011). Achievement of lipid targets with the combination of rosuvastatin and fenofibric Acid in patients with type 2 diabetes mellitus. Cardiovasc Drugs Ther.

[58] Ballantyne CM, Jones PH, Kelly MT, Setze CM, Lele A, Thakker KM, Stolzenbach JC (2011). Long-term efficacy of adding fenofibric acid to moderate-dose statin therapy in patients with persistent elevated triglycerides. Cardiovasc Drugs Ther.

[59] Davidson MH, Rooney MW, Drucker J, Eugene Griffin H, Oosman S, Beckert M (2009). Efficacy and tolerability of atorvastatin/fenofibrate fixed-dose combination tablet compared with atorvastatin and fenofibrate monotherapies in patients with dyslipidemia: a 12-week, multicenter, double-blind, randomized, parallel-group study. Clin Ther.

[60] Sacks FM, Carey VJ, Fruchart JC (2010). Combination lipid therapy in type 2 diabetes. N Engl J Med.

[61] Jun M, Foote C, Lv J, Neal B, Patel A, Nicholls SJ, Grobbee DE (2010). Effects of fibrates on cardiovascular outcomes: a systematic review and meta-analysis. Lancet.

[62] Bruckert E, Labreuche J, Deplanque D, Touboul PJ, Amarenco P (2011). Fibrates effect on cardiovascular risk is greater in patients with high triglyceride levels or atherogenic dyslipidemia profile: a systematic review and meta-analysis. J Cardiovasc Pharmacol.

